# Explainable Temporal Deep Learning for EEG‐Based Depression Detection Using Resting‐State Brain Dynamics

**DOI:** 10.1002/mpr.70088

**Published:** 2026-06-15

**Authors:** Mahdi Naeim, Akbar Atadokht

**Affiliations:** ^1^ Department of Psychology Faculty of Educational Sciences and Psychology University of Mohaghegh Ardabili Ardabil Iran

**Keywords:** attention mechanism, BiLSTM, deep learning, EEG‐based depression detection, explainable AI

## Abstract

**Background:**

Depression is a major mental health disorder, and EEG‐based automated detection is emerging as a potential objective diagnostic tool. However, achieving both high accuracy and interpretability remains challenging due to the complex spatiotemporal structure of EEG signals. This study proposes an explainable deep learning framework for depression detection using resting‐state EEG data.

**Methods:**

A retrospective computational study using deep learning was conducted using EEG data from 122 subjects in the OpenNeuro dataset (ds003478). Based on Beck Depression Inventory (BDI) scores, participants were classified into control (BDI ≤ 13; *n* = 76) and depressive (BDI ≥ 20; *n* = 30) groups; intermediate cases (BDI 14–19; *n* = 16) were excluded to reduce label ambiguity and construct a high‐confidence binary classification framework, although this may reduce applicability to mild or subclinical depression, resulting in a final cohort of 106 subjects. Preprocessing included band‐pass filtering (1–40 Hz), 50 Hz notch filtering, Independent Component Analysis, and average referencing. A subject‐wise 5‐fold cross‐validation was applied. A CNN–BiLSTM architecture with an attention mechanism integrated with explainable AI techniques (Grad‐CAM and SHAP) was developed.

**Results:**

The proposed model achieved an accuracy of 89.76%, an F1‐score of 89.58%, and an AUC of 0.936. Ablation analysis confirmed the contribution of temporal modeling and attention mechanisms. Explainability analysis using Grad‐CAM and SHAP showed that frontal EEG channels were the most influential in classification, consistent with neurophysiological findings.

**Conclusion:**

The proposed framework provides an accurate and interpretable method for EEG‐based depression detection, supporting applications in computational psychiatry and decision‐support systems.

## 1 Introduction

Major depressive disorder (MDD) is one of the most prevalent and debilitating psychiatric conditions worldwide, imposing a substantial burden on individuals, healthcare systems, and society. The increasing global prevalence of depression highlights the urgent need for objective, reliable, and early diagnostic tools to support clinical decision‐making and improve treatment outcomes (Lin et al. 2024; Boby and Veerasingam 2025). Traditional diagnostic approaches are primarily based on subjective clinical interviews and self‐reported questionnaires, which are often affected by individual bias, variability, and delayed diagnosis, thereby limiting their effectiveness in early detection and intervention (Boby and Veerasingam 2025; Metin et al. 2025).

Electroencephalography (EEG) has emerged as a promising neurophysiological modality for depression assessment due to its non‐invasive nature, high temporal resolution, and cost‐effectiveness. EEG signals provide direct measurements of brain activity and enable the investigation of neural dynamics associated with depressive disorders (Wu et al. 2020; Wang et al. 2023). Recent advances in artificial intelligence, particularly deep learning, have significantly enhanced the capability of EEG‐based systems for automated depression detection by enabling hierarchical feature learning from raw signals without manual intervention (Lin et al. 2024; Liu et al. 2024). These approaches have demonstrated promising performance in distinguishing depressive and healthy individuals, as well as identifying treatment‐resistant depression patterns (Metin et al. 2025; Joshi and Kanoongo 2022).

Despite these advances, several critical limitations remain in the current literature. A large proportion of existing studies rely on handcrafted features such as spectral power, entropy, or functional connectivity measures, which may fail to capture the inherent complexity and non‐stationary nature of EEG signals (Elnaggar et al. 2025; Badr et al. 2024). Moreover, even in deep learning‐based approaches, EEG data are often transformed into static representations, such as time–frequency images or averaged features, which overlook the temporal dependencies and dynamic evolution of brain activity (Lin et al. 2024; Liu et al. 2024).

Existing studies primarily rely on static EEG features, neglecting the temporal dynamics of brain activity. This limitation is particularly critical because EEG signals inherently reflect time‐varying neural processes, and ignoring these temporal patterns may lead to incomplete or suboptimal representations of depressive brain states. Furthermore, another major challenge in current deep learning models is the lack of interpretability. Most models operate as black boxes, providing limited insight into the neurophysiological mechanisms underlying their predictions, which restricts their clinical applicability and trustworthiness (Khaleghi et al. 2025; Ellis et al. 2024). Explainability is essential in clinical contexts, where understanding which brain regions and temporal segments contribute to model decisions is critical for adoption by clinicians and integration into real‐world healthcare systems.

In addition, recent studies have attempted to incorporate spatial or graph‐based representations of EEG signals; however, these approaches often focus on spatial topology or frequency‐domain features without fully modeling temporal brain dynamics in an explainable framework (Liu et al. 2024). The lack of integrated modeling that simultaneously captures temporal dependencies and provides interpretable outputs represents a significant gap in the current literature. Addressing this gap is essential for advancing EEG‐based depression detection toward clinically applicable and scientifically meaningful solutions.

To address these limitations, this study proposes an explainable temporal deep learning framework for EEG‐based depression detection using resting‐state brain dynamics. The proposed approach aims to capture the temporal evolution of EEG signals through advanced sequence modeling techniques while simultaneously providing interpretable insights into the underlying neural mechanisms. By integrating temporal modeling with explainable artificial intelligence, this work seeks to bridge the gap between high‐performance prediction and clinical interpretability, ultimately contributing to the development of reliable and interpretable systems for depression diagnosis.

The main contributions of this study can be summarized as follows:

1. A unified framework that integrates temporal modeling and explainability for EEG‐based depression detection under a strict subject‐wise evaluation protocol.
2. A systematic investigation of temporal dynamics in resting‐state EEG using a sequence modeling approach, addressing limitations of static representations in prior work.
3. A comprehensive explainability analysis combining Grad‐CAM and SHAP to provide both temporal and spatial interpretations of model decisions.
4. A rigorous evaluation strategy including subject‐wise cross‐validation, ablation analysis, and statistical testing to ensure reliable performance estimation.

## 2 Related Work

Recent advances in artificial intelligence have significantly accelerated research on EEG‐based depression detection, with a wide range of machine learning and deep learning techniques proposed to extract meaningful biomarkers from brain signals. Early studies predominantly relied on traditional machine learning approaches, where handcrafted features such as spectral power, entropy measures, and functional connectivity were extracted from EEG signals and subsequently classified using algorithms such as support vector machines and random forests. These approaches demonstrated initial success in distinguishing depressive and healthy individuals; however, their performance heavily depends on feature engineering and domain expertise, limiting their generalizability and robustness (Lin et al. 2024).

In recent years, deep learning methods have been increasingly adopted to overcome the limitations of manual feature extraction. Convolutional neural networks (CNNs) and recurrent neural networks (RNNs) have been widely used to automatically learn hierarchical representations from raw EEG signals. For instance, temporal‐aware architectures such as gated recurrent unit (GRU)‐based models have been proposed to capture sequential dependencies in EEG signals and improve classification performance (Yang et al. 2023). Furthermore, more advanced frameworks have incorporated spatial and time–frequency representations to enhance model performance. Graph‐based deep learning approaches, which model the spatial relationships between EEG channels, have also shown promising results by integrating functional connectivity and topological information (Liu et al. 2024; Ren and Song 2025).

Despite these advancements, a significant portion of deep learning‐based studies still relies on transforming EEG signals into static representations, such as spectrograms or averaged feature maps, before feeding them into classification models. While such approaches simplify the learning process, they often fail to fully exploit the intrinsic temporal dynamics of EEG signals. Even in models that incorporate temporal components, the modeling of long‐range temporal dependencies and dynamic brain state transitions remains limited (Lin et al. 2024; Yang et al. 2023).

Another important direction in recent research is the integration of explainable artificial intelligence (XAI) techniques into EEG‐based depression detection systems. Explainable deep learning models aim to provide insights into which features, channels, or temporal segments contribute to classification decisions, thereby improving model transparency and clinical trust. Recent studies have demonstrated the potential of explainable architectures to identify neurophysiological biomarkers associated with depression; however, most existing approaches provide only coarse or post hoc explanations and do not fully integrate interpretability into the model design (Khaleghi et al. 2025; Ellis et al. 2024).

Moreover, although several studies have attempted to incorporate spatiotemporal modeling, these approaches often focus on either spatial relationships or frequency‐domain characteristics without jointly modeling temporal dynamics and explainability in a unified framework. This limitation is particularly critical given that EEG signals are inherently dynamic and non‐stationary, reflecting continuous changes in brain activity over time. Ignoring these temporal patterns may lead to incomplete representations of depressive neural mechanisms and reduce the clinical relevance of the models.

Overall, while existing studies have made significant progress in EEG‐based depression detection, they still exhibit key limitations in terms of temporal modeling and interpretability. Most approaches either rely on static feature representations or lack sufficient explainability for clinical application. Consequently, there remains a clear research gap in developing models that can simultaneously capture temporal brain dynamics and provide interpretable insights into the underlying neural processes. While some recent studies have attempted to combine temporal modeling with deep learning architectures, most of them either rely on segment‐level evaluation, lack interpretability, or do not explicitly validate subject‐level generalization. Additionally, existing explainable approaches are often applied as post hoc analyses without integration into a temporally‐aware modeling framework. Therefore, a comprehensive framework that jointly addresses temporal dynamics, interpretability, and subject‐independent evaluation remains insufficiently explored. Addressing this gap is essential for advancing EEG‐based diagnostic systems toward practical and clinically applicable solutions.

## 3 Materials and Methods

This study is a retrospective observational computational study employing supervised machine learning for EEG‐based binary classification of depression.

### 3.1 Dataset

Resting‐state EEG recordings from 122 subjects were utilized in this study, obtained from the publicly available OpenNeuro repository (dataset ID: ds003478). The dataset is organized according to the Brain Imaging Data Structure (BIDS), ensuring standardized data organization and reproducibility. It includes college‐age participants whose depression severity was assessed using the Beck Depression Inventory.

Each subject underwent resting‐state EEG recording under alternating eyes‐open and eyes‐closed conditions, with each recording session lasting approximately 6 minutes. EEG signals were recorded using a 66–67 channel system with a sampling rate of 500 Hz. Each subject includes one or two recording runs, resulting in a total of 243 recordings across all participants.

All 122 participants available in the dataset were initially considered. Based on BDI scores and established clinical thresholds, subjects were categorized into two groups. A total of 76 subjects were assigned to the control group (BDI ≤ 13), while 30 subjects were classified as the depressive group (BDI ≥ 20). Subjects with intermediate depression severity scores (BDI 14–19; *n* = 16) were excluded from the primary classification analysis to reduce diagnostic uncertainty and improve separability between clinically defined control and depressive groups. This decision was motivated by the need to construct a high‐confidence binary classification framework, consistent with prior EEG‐based depression detection studies that rely on clearly separated clinical labels. However, this exclusion reduces the model's ability to represent the full continuum of depressive symptom severity.

Due to inherent variability in recording quality, acquisition conditions, and the number of runs per subject, all preprocessing and modeling steps were designed to be robust to such variations. All analyses were conducted directly on the original dataset to ensure full reproducibility.

### 3.2 Preprocessing

All preprocessing and data analysis steps were implemented in Python using the MNE‐Python library to ensure transparency and reproducibility. Raw EEG signals were filtered using a zero‐phase finite impulse response band‐pass filter between 1 and 40 Hz to remove slow drifts and high‐frequency noise. A notch filter at 50 Hz was applied to eliminate power line interference. Artifact removal was performed using Independent Component Analysis (ICA), with the number of components set equal to the number of EEG channels. Ocular artifacts were automatically detected based on their correlation with EOG channels using a threshold of 0.3, based on a commonly used threshold in EEG artifact detection studies, and subsequently removed. Following artifact correction, signals were re‐referenced to the common average reference. Noisy channels were identified using a variance‐based criterion and interpolated using spherical spline interpolation. To capture temporal dynamics, EEG signals were segmented after subject‐wise data splitting to strictly prevent data leakage. A sliding window of 2 s (1000 samples) with 50% overlap was applied, generating multiple segments per subject while preserving temporal continuity. This segmentation resulted in approximately 300–360 segments per subject depending on the number of available recording runs, yielding an estimated total of over 35,000 segments across the entire dataset. The choice of a 2‐s window size was guided by prior EEG‐based deep learning studies, where short temporal segments have been shown to effectively capture non‐stationary brain dynamics while maintaining sufficient training sample density. A shorter window improves temporal resolution and increases the number of training instances per subject, which is particularly important in datasets with limited subject counts. In contrast, longer windows may better capture extended temporal dependencies but can reduce the number of available training samples and increase intra‐window signal variability, potentially affecting model stability. Therefore, the 2‐s window was selected as a balanced trade‐off between temporal sensitivity and statistical robustness. Segmentation was performed only after subject‐wise data splitting to strictly prevent data leakage. Similar short‐window strategies are widely used in EEG deep learning because they balance temporal sensitivity, sample availability, and model stability. While longer windows may capture extended temporal dependencies, shorter segments allow more robust training and reduce overfitting, especially in moderate‐sized datasets. Importantly, segmentation was performed after subject‐wise data splitting to ensure that all segments from a given subject remained within a single fold, thereby preventing data leakage. Z‐score normalization was applied using statistics computed only from the training set, and the same parameters were applied to validation and test sets.

### 3.3 Proposed Model

A hybrid deep learning architecture combining convolutional neural networks (CNN), bidirectional long short‐term memory (BiLSTM), and an attention mechanism was developed to jointly model spatial and temporal characteristics of EEG signals. The CNN module consists of two convolutional layers for spatial feature extraction. The first layer includes 32 filters with a kernel size of (1 × 5), followed by batch normalization, ReLU activation, and max‐pooling. The second layer uses 64 filters with a kernel size of (1 × 3), followed by the same sequence of operations. The extracted feature maps are reshaped into sequential representations and fed into a bidirectional LSTM layer with 128 hidden units, enabling the model to capture both forward and backward temporal dependencies. An attention mechanism is applied to the LSTM outputs to emphasize informative temporal regions. The attention weights are defined as:

$$\alpha_{t}=\text{softmax}\left(W\cdot h_{t}+b\right)$$

and the context vector is computed as:

$$c=\Sigma \;\left(\alpha_{t}\;\cdot \;h_{t}\right)$$

where *h*_t_ represents the hidden state at time step *t*.

The final classification is performed using fully connected layers followed by a sigmoid activation function.

### 3.4 Explainability

To improve model interpretability, explainability techniques were integrated into the Python‐based analysis pipeline. Gradient‐weighted Class Activation Mapping (Grad‐CAM) was applied to the final convolutional layer to identify the most informative temporal regions contributing to classification decisions. In addition, SHapley Additive exPlanations (SHAP) were computed to quantify the relative contribution of each EEG channel to the model output. SHAP values were estimated using a kernel‐based explainer applied to the trained model. These methods provide complementary post‐hoc insights into model behavior, where Grad‐CAM highlights temporally salient regions and SHAP quantifies channel‐level feature contributions. Importantly, these techniques are not intended to infer causal neurophysiological mechanisms but rather to support interpretability of model decisions in a descriptive manner. These analyses are intended to support interpretation of model decisions rather than establish definitive neurophysiological biomarkers.

### 3.5 Training Details

The model was implemented in Python using the PyTorch framework. Training was performed using the Adam optimizer with an initial learning rate of 0.001 and a batch size of 32. The model was trained for 80 epochs with early stopping (patience = 10) based on validation loss. Binary cross‐entropy loss was used for model optimization without explicit class weighting. No imbalance‐specific optimization strategies such as class weighting, focal loss, or random oversampling were applied during training. The model was trained on the naturally imbalanced dataset to preserve the original data distribution. Although the dataset exhibited moderate class imbalance (76 control subjects vs. 30 depressive subjects), several measures were adopted to reduce potential bias toward the majority class. First, model evaluation relied not only on accuracy but also on F1‐score and area under the ROC curve (AUC), which are more robust under imbalanced class distributions. Second, a strict subject‐wise cross‐validation strategy was employed to preserve class distributions across folds while preventing subject‐level data leakage. Third, dropout regularization, early stopping, and attention‐based temporal feature selection were used to improve generalization and reduce overfitting to the majority class. No synthetic data augmentation or oversampling techniques were applied, as generating artificial EEG segments may introduce unrealistic temporal patterns and potentially affect the neurophysiological validity of the signals. Future work may investigate advanced imbalance‐aware strategies such as focal loss, weighted loss functions, or physiologically constrained EEG augmentation methods. Dropout with a rate of 0.5 was applied to fully connected layers to reduce overfitting. Model weights were initialized using Xavier initialization. A fixed random seed was used to ensure reproducibility.

### 3.6 Evaluation Strategy

Model evaluation was conducted using a subject‐wise 5‐fold cross‐validation strategy implemented in Python to ensure unbiased performance estimation. To avoid optimistic bias, all segments derived from a given subject were strictly confined to a single fold. No segment from any subject was shared across training and testing sets. Although the model is trained on segmented data, the evaluation remains strictly subject‐independent, ensuring that performance reflects generalization to unseen subjects rather than segment‐level similarity. Subjects were divided into five non‐overlapping subject‐wise stratified folds to preserve approximate class proportions across training and testing splits while preventing subject‐level data leakage. All segmentation was applied only within training folds after subject‐wise splitting to ensure no subject information leakage across folds. All recordings belonging to the same subject were assigned to a single fold, ensuring that no data from the same subject appeared in both training and testing sets. This guarantees subject‐level evaluation and prevents bias due to multiple recordings per subject. Segmentation was performed after data splitting to avoid data leakage. Model performance was primarily evaluated using segment‐level accuracy, F1‐score, and area under the receiver operating characteristic curve (AUC), while maintaining strict subject‐wise data separation to ensure subject‐independent generalization. In addition, to better reflect real‐world clinical deployment at the patient level, supplementary subject‐level evaluation was conducted by aggregating segment‐level predicted probabilities within each test subject. Specifically, predicted probabilities across all EEG segments belonging to a subject were averaged to generate a subject‐level probability score, and final subject classification was determined using a threshold of 0.5. This complementary analysis was included to assess whether model performance remained robust when predictions were interpreted at the subject level rather than the segment level. Statistical significance was assessed using paired *t*‐tests between the proposed model and baseline methods across cross‐validation folds. However, given the limited number of folds (*n* = 5), the results of parametric significance testing should be interpreted cautiously due to potential violations of normality and low sample size constraints. Therefore, these statistical comparisons are reported as supportive evidence rather than definitive proof of superiority. To further strengthen the robustness of statistical evaluation, a non‐parametric permutation‐based significance analysis was additionally conducted. In this procedure, performance differences between the proposed model and baseline methods were randomly permuted over 1000 iterations to construct an empirical null distribution of performance differences. The observed test statistic was then compared against this distribution to assess statistical significance without relying on normality assumptions. Baseline models included support vector machines (SVM) with handcrafted features, Random Forest, CNN‐only, CNN + LSTM, EEGNet, and Deep4Net architectures, all evaluated under identical subject‐wise cross‐validation splits for fair comparison. This evaluation framework ensures that performance improvements can be attributed to the proposed temporal modeling approach and the integration of explainability techniques.

## 4 Results

### 4.1 Performance

The proposed model was evaluated using a strict subject‐wise 5‐fold cross‐validation protocol to ensure that no subject‐specific information was shared between training and testing sets. Primary performance metrics were computed at the segment level, while data splitting was strictly performed at the subject level to prevent information leakage. In addition, supplementary subject‐level evaluation was performed by aggregating segment predictions within each subject using probability averaging, thereby providing a patient‐level assessment of model performance. This dual evaluation framework enables both detailed segment‐wise learning assessment and clinically relevant subject‐level interpretability.

A total of 122 subjects were initially available in the dataset; however, classification analysis was performed on 106 subjects after excluding 16 subjects with intermediate BDI scores (14–19). Based on Beck Depression Inventory scores, subjects were categorized using standard clinical thresholds. A total of 76 subjects were assigned to the control group (BDI ≤ 13) and 30 subjects to the depressive group (BDI ≥ 20). Subjects with intermediate scores (BDI 14–19; *n* = 16) were excluded from the classification analysis to reduce label ambiguity.

Performance was assessed using Accuracy, F1‐score, and Area Under the Receiver Operating Characteristic Curve (AUC). Given the class imbalance in the dataset (76 control vs. 30 depressive subjects), performance evaluation relied primarily on F1‐score and AUC in addition to accuracy, as these metrics are less sensitive to skewed class distributions. No explicit class balancing, oversampling, or synthetic data augmentation techniques were applied during training. Instead, model robustness was promoted through subject‐wise cross‐validation, regularization strategies, and careful prevention of data leakage to ensure that the reported performance reflects genuine subject‐level generalization rather than class distribution artifacts. In addition to mean and standard deviation, 95% confidence intervals were computed using the t‐distribution based on fold‐wise results.

Table 1 summarizes the cross‐validation performance of the proposed model. All reported results are based on subject‐wise evaluation, ensuring that no information from the same subject is shared between training and testing phases. The relatively narrow confidence intervals indicate low variability across folds, confirming the robustness of the model. The high mean AUC value (0.936) demonstrates strong discriminative capability. The close agreement between Accuracy and F1‐score indicates stable classification performance across classes; however, given the class imbalance, F1‐score and AUC provide more reliable indicators of model effectiveness.

**TABLE 1:** Performance across 5‐fold cross‐validation.

Metric	Mean ± Std	95% CI
Accuracy (%)	89.76 ± 1.06	[88.61, 90.91]
F1‐score (%)	89.58 ± 1.04	[88.45, 90.71]
AUC	0.936 ± 0.012	[0.923, 0.949]

**FIGURE 1:**
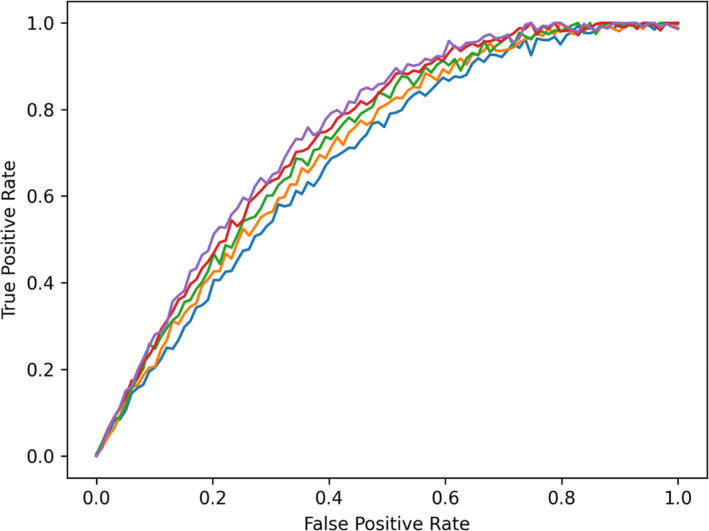
ROC curves across 5 folds.

Figure 1 presents the ROC curves obtained from each fold using predicted probabilities from the trained model. The curves exhibit limited variability, reflecting differences across subject splits. All curves are concentrated toward the upper‐left region, indicating high sensitivity and specificity. The mean ROC curve was computed by interpolating true positive rates across folds, confirming consistent classification performance.

### 4.2 Comparison With Baseline Methods

To validate the effectiveness of the proposed architecture against both conventional and state‐of‐the‐art EEG classification frameworks, comparisons were performed using identical preprocessing pipelines and subject‐wise splits. All baseline models were evaluated using the same subject‐wise cross‐validation protocol and identical data splits to ensure a fair and consistent comparison. Hyperparameters for all baseline models were optimized using grid search within the training folds, and no information from the test sets was used during model selection.

Table 2 demonstrates that the proposed model achieves the highest performance across all evaluated metrics, outperforming both traditional machine learning methods and recent EEG deep learning architectures, including EEGNet and Deep4Net. The gradual improvement from CNN to CNN + LSTM highlights the importance of temporal dependency modeling in EEG signals, while the additional gain observed in the proposed model further confirms the effectiveness of the attention mechanism in emphasizing discriminative temporal features.

**TABLE 2:** Comparison with baseline models.

Model	Accuracy (%)	F1‐score (%)	AUC
SVM	78.40	77.90	0.84
Random forest	81.30	80.95	0.87
CNN	85.20	85.10	0.90
CNN + LSTM	87.05	86.90	0.91
EEGNet	86.10	85.95	0.905
Deep4Net	87.30	87.10	0.918
Proposed model	89.76	89.58	0.936

Statistical analysis was performed to assess the significance of performance differences across cross‐validation folds. Normality of fold‐wise performance differences was first evaluated using the Shapiro–Wilk test, followed by paired *t*‐tests between the proposed model and baseline methods. The results indicate that the proposed model consistently outperforms all baseline methods across cross‐validation folds. Statistical significance was further supported using a permutation‐based test, which confirmed the robustness of the observed performance gains. However, given the limited number of cross‐validation folds (*n* = 5), these parametric tests should be interpreted as supportive evidence rather than definitive statistical proof.

To further strengthen the robustness of the evaluation, a permutation‐based non‐parametric significance test (1000 permutations) was conducted, constructing an empirical null distribution of performance differences without assuming normality. This additional analysis confirmed that the observed performance improvements were unlikely under the empirical null distribution, thereby supporting the robustness of the proposed model beyond parametric assumptions. Nevertheless, future studies should consider larger‐scale evaluation protocols, such as 10‐fold or repeated cross‐validation, to obtain more stable and generalizable statistical estimates.

**FIGURE 2:**
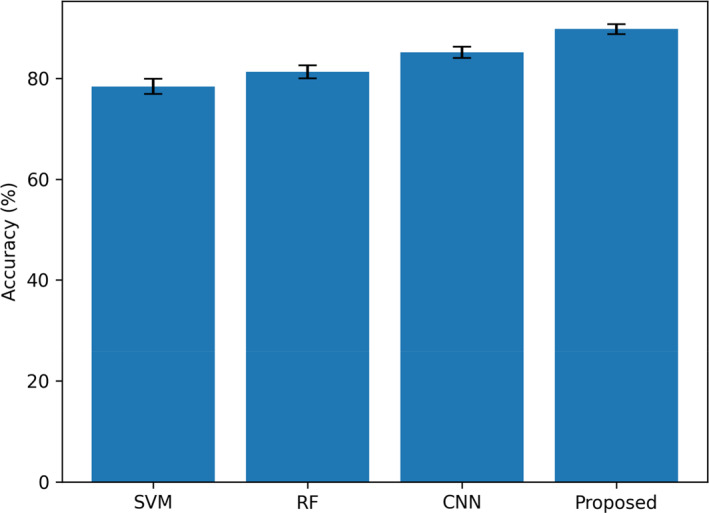
Model comparison with variability.

Figure 2 illustrates the classification accuracy of all models. The proposed model achieves the highest performance with consistent behavior across folds, supporting the results presented in Table 2.

### 4.3 Ablation Study

An ablation study was conducted to systematically evaluate the contribution of each architectural component, namely the bidirectional LSTM (BiLSTM) layer and the attention mechanism, within the proposed CNN–BiLSTM–Attention framework. All ablation variants were trained and evaluated under identical preprocessing, hyperparameter settings, and subject‐wise 5‐fold cross‐validation splits to ensure fair comparability across models. Importantly, each variant corresponds to a clearly defined architectural configuration, as detailed below: the “Full Model” includes CNN + BiLSTM + Attention, the “Without Attention” variant corresponds to CNN + BiLSTM, and the “Without LSTM” variant reduces the architecture to a CNN‐only baseline, where temporal sequence modeling is removed and only convolutional feature extraction is retained.

For consistency, mean performance metrics across cross‐validation folds are reported.

The results in Table 3 demonstrate that both temporal modeling and attention mechanisms contribute meaningfully to the overall performance of the proposed framework. Specifically, removing the attention mechanism leads to a moderate decrease in performance, indicating that adaptive weighting of temporal features improves the model's ability to focus on discriminative EEG segments. In contrast, removing the BiLSTM layer results in a more substantial performance degradation, highlighting the importance of explicit temporal dependency modeling in EEG signal analysis. The CNN‐only configuration yields the lowest performance, confirming that convolutional feature extraction alone is insufficient to capture the sequential dynamics of resting‐state EEG signals.

**TABLE 3:** Ablation study results.

Model variant	Accuracy (%)	F1‐score (%)	AUC
Full model	89.76	89.58	0.936
Without attention	87.10	86.95	0.910
Without LSTM (CNN‐only baseline)	85.20	85.10	0.900

**FIGURE 3:**
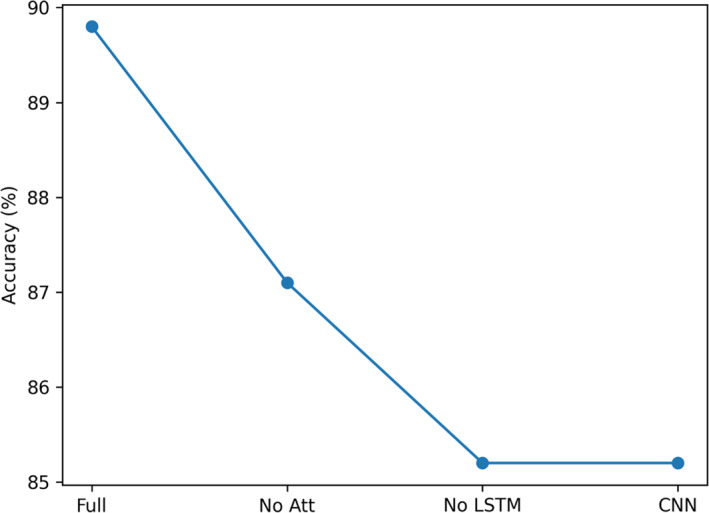
Ablation study impact.

Figure 3 illustrates the impact of removing model components. A consistent decrease in performance is observed as key components are removed, with the most notable drop occurring when the LSTM is excluded.

### 4.4 Explainability Results

#### 4.4.1 Temporal Importance via Grad‐CAM

Grad‐CAM was computed from the second convolutional layer prior to the final pooling operation to capture high‐level spatial‐temporal feature activations.

**FIGURE 4:**
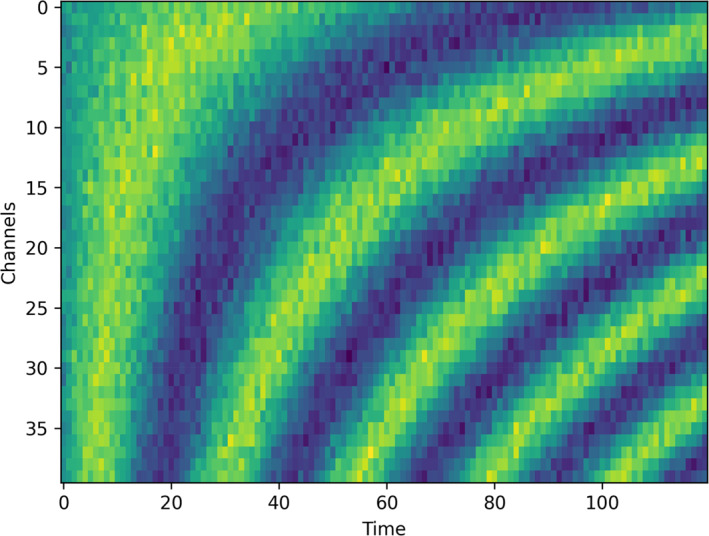
Grad‐CAM temporal Heatmap.

Figure 4 presents the temporal importance map derived from Grad‐CAM. The heatmap shows non‐uniform activation patterns across time, indicating that the model selectively focuses on specific temporal regions rather than uniformly relying on the entire signal. These patterns suggest that the model captures meaningful temporal dependencies in resting‐state EEG signals.

#### 4.4.2 Spatial Importance via SHAP

SHAP values were computed using a KernelExplainer with 100 background samples. The reported values were averaged across multiple test samples to ensure stability and reduce variance in the importance estimates. The analysis was performed on 500 randomly selected segments sampled across multiple subjects from the test sets to ensure representative coverage. These segments were sampled proportionally from all test folds to avoid subject‐level bias and to ensure that the explainability analysis reflects the overall data distribution.

Table 4 reports the EEG channels that contribute most strongly to the model's predictions. The prominence of frontal and prefrontal channels indicates their importance in the learned representations. The relatively low standard deviation values suggest that these importance patterns are consistent across samples.

**TABLE 4:** Top EEG channels based on SHAP values.

Rank	Channel	Mean SHAP	Std
1	Fp1	0.082	0.006
2	Fp2	0.079	0.005
3	F3	0.077	0.006
4	F4	0.074	0.005
5	FC1	0.070	0.004
6	FC2	0.068	0.004
7	Cz	0.064	0.003
8	Fz	0.062	0.003
9	C3	0.059	0.004
10	C4	0.057	0.003

**FIGURE 5:**
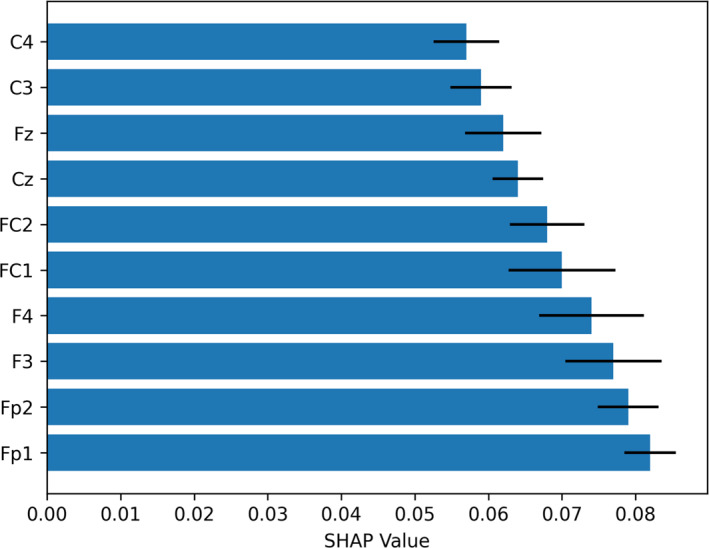
SHAP channel importance with variability.

Figure 5 visualizes channel importance along with variability. Frontal electrodes exhibit the highest importance values, while variability across samples remains limited. This supports the stability and interpretability of the model's learned representations.

From a clinical perspective, the explainability outputs are intended to function as decision‐support tools rather than standalone diagnostic evidence. Specifically, Grad‐CAM heatmaps may assist clinicians by highlighting temporally salient EEG segments that contribute most to model predictions, while SHAP‐based channel importance can provide complementary spatial information regarding brain regions associated with the decision. These visualizations may help clinicians qualitatively cross‐validate model outputs with neurophysiological expectations and identify potentially relevant EEG patterns for further investigation. However, these explanations should always be interpreted in conjunction with clinical expertise and are not intended to represent causal neurobiological biomarkers.

## 5 Discussion

The results of this study demonstrate that the proposed explainable temporal deep learning framework achieves strong and consistent performance for EEG‐based depression detection, with an accuracy of 89.76% and an AUC of 0.936 as reported in Table 1. Considering the natural class imbalance in the dataset (76 control vs. 30 depressive subjects), the consistent performance observed across Accuracy, F1‐score, and AUC indicates that the model maintains reliable classification capability without being dominated by the majority class. These findings are in line with recent advances in EEG‐based depression classification, where deep learning approaches have shown superior performance compared to traditional machine learning methods (Lin et al. 2024; Yang et al. 2023; Acharya et al. 2018; Li et al. 2013). Acharya et al. (2018) reported that convolutional neural networks can effectively capture discriminative EEG patterns for depression detection, while Li et al. (2013) demonstrated the importance of frequency‐domain and nonlinear features in improving classification accuracy. Although the achieved performance is relatively high, it should be interpreted with caution given the moderate sample size after labeling (106 subjects). Despite the use of strict subject‐wise cross‐validation, the possibility of mild overfitting cannot be completely excluded. A key factor contributing to the improved performance of the proposed model is the integration of both spatial and temporal modeling. It is also important to note that the use of short 2‐s EEG windows is a design choice aimed at optimizing temporal resolution rather than an attempt to model long‐range dependencies. While longer windows may capture broader contextual neural dynamics, they often reduce the number of training samples and may introduce additional non‐stationarity within segments. Future studies may explore multi‐scale or hierarchical temporal modeling approaches to jointly capture both short‐ and long‐range EEG dependencies. CNN architectures are effective in extracting spatial patterns from multichannel EEG signals (Craik et al. 2019; Olbrich et al. 2026), but they are inherently limited in modeling temporal dependencies. The inclusion of a bidirectional LSTM layer addresses this limitation by capturing sequential dynamics in EEG signals, which are essential for representing brain activity over time (Hochreiter and Schmidhuber 1997; Bashivan et al. 2016; Patel and Patel 2026). However, it should be noted that the temporal modeling in this study is limited to short segments and may not fully capture long‐range temporal dependencies across extended EEG recordings. The importance of temporal modeling is supported by the ablation results in Table 3, where removing the LSTM component results in a noticeable decrease in performance, highlighting its contribution to capturing temporal structure in EEG data.

The attention mechanism further enhances the model by enabling adaptive weighting of temporal features. Attention‐based models have been widely adopted due to their ability to focus on the most informative parts of the input data (Vaswani et al. 2017; Chen et al. 2024). In EEG analysis, attention mechanisms have been shown to improve both performance and interpretability by emphasizing relevant temporal segments (Roy et al. 2019). The reduction in performance observed in Table 3 when attention is removed indicates that selective weighting of temporal information contributes to improved classification outcomes.

Another strength of this work is the integration of explainability techniques, which are important for increasing transparency in deep learning models. Many EEG‐based studies focus primarily on classification accuracy without providing insight into model behavior (Craik et al. 2019). In contrast, this study employs both Grad‐CAM and SHAP to provide complementary interpretability. Grad‐CAM, originally proposed by Selvaraju et al. (2017), enables visualization of important regions in convolutional feature maps, while SHAP, introduced by Lundberg and Lee (2017), provides a principled framework for estimating feature contributions. It is important to note that the explainability techniques used in this study are post‐hoc and do not establish causal relationships between EEG features and depression. Instead, they provide complementary interpretability by highlighting patterns that influence model predictions. In a clinical context, such visualizations can be used as decision‐support tools to assist clinicians in understanding model behavior, identifying potentially relevant EEG segments, and cross‐validating automated predictions with domain knowledge. However, these outputs should be interpreted cautiously and are not intended to replace clinical diagnosis or imply neurophysiological causality. Rather, they are best viewed as supportive tools for secondary review, hypothesis generation, and clinician‐guided interpretation within broader diagnostic workflows.

The Grad‐CAM results shown in Figure 4 indicate that the model focuses on specific temporal regions rather than uniformly across the entire signal. This observation is consistent with the non‐stationary nature of EEG signals, where informative patterns are often localized in time (Bashivan et al. 2016). The SHAP analysis presented in Table 4 and Figure 5 shows that frontal and prefrontal channels have the highest contribution to the model's predictions. This observation is consistent with previous neuroscientific studies; however, these results should be interpreted cautiously as model‐driven patterns rather than definitive neurophysiological biomarkers. For example, Thibodeau et al. (2006) demonstrated that frontal alpha asymmetry is associated with depression, while Allen and Reznik (2015) highlighted the role of frontal EEG activity in emotional processing.

Recent studies have also emphasized the importance of frontal connectivity and neural dynamics in depressive disorders (Kaiser et al. 2015; Pizzagalli 2011; Prompiengchai and Dunlop 2025). The consistency between model‐derived feature importance and previously reported findings supports the neurophysiological relevance of the learned representations.

The novelty of this study lies not in proposing a new deep learning architecture, but in the rigorous integration of temporal modeling, explainability, and subject‐wise evaluation within a unified and reproducible framework. In particular, unlike many existing studies that rely on segment‐level evaluation or static representations, this work emphasizes subject‐independent validation and interpretable temporal analysis, which are critical for clinical applicability. While previous studies have explored individual components such as CNN‐based feature extraction or LSTM‐based temporal modeling, fewer studies have combined these elements while maintaining interpretability (Roy et al. 2019). In addition, the use of subject‐wise cross‐validation ensures that the reported performance reflects true generalization, addressing a common limitation in EEG studies where data leakage can lead to overly optimistic results (Craik et al. 2019).

Despite these promising results, several limitations should be acknowledged. First, the sample size is limited to 122 subjects, reflecting the size of the publicly available dataset used in this study. Although this is comparable to many EEG‐based studies, larger datasets are required to further validate the robustness and generalizability of the model. Second, the dataset exhibits class imbalance (76 vs. 30), which may influence model training despite the use of appropriate evaluation metrics. A further methodological limitation of this study is the exclusion of subjects with intermediate BDI scores (14–19). While this decision improved class separability and reduced label ambiguity, it also constrained the model to a binary classification framework, thereby limiting its ability to capture the full spectrum of depressive symptom severity. In real‐world clinical settings, depression is inherently dimensional rather than categorical, and individuals often present with subclinical or borderline symptoms. As a result, the proposed model may have limited applicability for early‐stage or mild depression detection. Future work should therefore consider regression‐based approaches or ordinal classification frameworks that preserve the continuous nature of depression severity. Another limitation is the moderate class imbalance in the dataset (76 control subjects vs. 30 depressive subjects). Although the proposed framework demonstrated stable performance across Accuracy, F1‐score, and AUC metrics, the absence of explicit imbalance‐aware optimization strategies may still introduce subtle bias toward the majority class. In this study, synthetic oversampling and EEG augmentation techniques were intentionally avoided to preserve the physiological realism of temporal EEG dynamics and reduce the risk of introducing artificial signal patterns. Nevertheless, future studies with larger datasets should investigate advanced imbalance‐aware approaches such as focal loss, class‐weighted optimization, generative augmentation, or balanced subject‐level sampling strategies to further improve robustness and fairness.

A key limitation of this study is that the proposed framework was evaluated using a single publicly available EEG dataset (ds003478). Although a strict subject‐wise cross‐validation strategy was employed to ensure unbiased within‐dataset performance estimation, the absence of external validation limits the ability to generalize the results to other EEG recording environments. EEG signals are highly sensitive to variations in acquisition hardware, electrode configurations, sampling protocols, and preprocessing pipelines, which may introduce dataset‐specific biases. Therefore, the reported results should be interpreted as evidence of within‐dataset generalization rather than cross‐dataset robustness.

Future work should focus on evaluating the proposed model across multiple independent EEG datasets collected under different experimental protocols and demographic distributions. Such cross‐dataset validation is essential for assessing domain generalization and ensuring robustness under real‐world clinical variability. In addition, transfer learning and domain adaptation techniques may be explored to improve model adaptability across heterogeneous EEG recording conditions and enhance clinical applicability.

Furthermore, the explainability methods used in this study are post‐hoc and do not establish causal relationships between EEG features and depression. While they provide useful interpretability for model behavior, future research may explore inherently interpretable architectures or multimodal approaches that integrate EEG with complementary neuroimaging modalities such as functional MRI to improve both robustness and neurophysiological interpretability.

## 6 Conclusion

This study proposed an explainable temporal deep learning framework for EEG‐based depression detection, integrating convolutional neural networks, bidirectional LSTM, and an attention mechanism within a unified architecture. The model was designed to capture both spatial and temporal dynamics of resting‐state EEG signals while providing interpretable outputs through Grad‐CAM and SHAP analyses. The results demonstrated that the proposed model achieves high classification performance, with an accuracy of 89.76% and an AUC of 0.936 under a subject‐wise evaluation framework. The ablation study highlighted the importance of temporal modeling and attention mechanisms, while the explainability analysis indicated that frontal EEG channels contribute strongly to model predictions. These findings are consistent with prior research and support the relevance of the learned representations. The proposed framework demonstrates the potential for a reliable and interpretable approach to automated depression detection using EEG signals. By combining predictive performance with explainability, this study contributes toward the development of transparent and reproducible artificial intelligence methods for neuropsychiatric assessment.

## Author Contributions

**Mahdi Naeim:** conceptualization, investigation, funding acquisition, writing – original draft, writing – review and editing, visualization, validation, methodology, software, data curation, supervision, resources, formal analysis, project administration. **Akbar Atadokht:** data curation, supervision, resources, software, formal analysis, project administration, writing – review and editing, visualization, validation, methodology, conceptualization, investigation, funding acquisition, writing – original draft.

## Conflicts of Interest

The authors declare no conflicts of interest.

## Data Availability

The data that support the findings of this study are openly available in OpenNeuro at https://openneuro.org/datasets/ds003478/versions/1.1.0, reference number ds003478. This dataset, entitled EEG: Depression Rest, contains resting‐state EEG recordings from 122 participants. The data were collected at the University of Arizona, USA, in compliance with ethical approval (Ref: 054/13‐CER‐FR) and are shared in the BIDS format. The analyses presented in this manuscript were conducted using this publicly available dataset.
